# Long-Term Persistence of Bi-functionality Contributes to the Robustness of Microbial Life through Exaptation

**DOI:** 10.1371/journal.pgen.1005836

**Published:** 2016-01-29

**Authors:** Maximilian G. Plach, Bernd Reisinger, Reinhard Sterner, Rainer Merkl

**Affiliations:** Institute of Biophysics and Physical Biochemistry, University of Regensburg, Regensburg, Germany; University of Michigan, UNITED STATES

## Abstract

Modern enzymes are highly optimized biocatalysts that process their substrates with extreme efficiency. Many enzymes catalyze more than one reaction; however, the persistence of such ambiguities, their consequences and evolutionary causes are largely unknown. As a paradigmatic case, we study the history of bi-functionality for a time span of approximately two billion years for the sugar isomerase HisA from histidine biosynthesis. To look back in time, we computationally reconstructed and experimentally characterized three HisA predecessors. We show that these ancient enzymes catalyze not only the HisA reaction but also the isomerization of a similar substrate, which is commonly processed by the isomerase TrpF in tryptophan biosynthesis. Moreover, we found that three modern-day HisA enzymes from Proteobacteria and Thermotogae also possess low TrpF activity. We conclude that this bi-functionality was conserved for at least two billion years, most likely without any evolutionary pressure. Although not actively selected for, this trait can become advantageous in the case of a gene loss. Such exaptation is exemplified by the Actinobacteria that have lost the *trp*F gene but possess the bi-functional HisA homolog PriA, which adopts the roles of both HisA and TrpF. Our findings demonstrate that bi-functionality can perpetuate in the absence of selection for very long time-spans.

## Author Summary

The term exaptation describes the process by which a trait that is initially just a by-product of another function may become important in a later evolutionary phase. For example, feathers served to insulate dinosaurs before helping birds fly. On the level of enzymes, bi-functionality can contribute to microbial evolution through exaptation. However, bi-functional enzymes may cause metabolic conflicts, if they are involved in different metabolic pathways. By characterizing properties of modern and computationally reconstructed ancestral variants of the sugar isomerase HisA, we demonstrate that it has been a bi-functional enzyme for the last two billion years. Most likely, bi-functionality persisted because the remaining TrpF activity is not harmful or its elimination would concurrently compromise HisA activity. Moreover, this substrate ambiguity is advantageous, as it allows compensating a gene loss as exemplified by the Actinobacteria. These microbes have lost the isomerase TrpF but possess the bi-functional HisA homolog PriA, which takes over the roles of both HisA and TrpF. Our results argue to view bi-functionality not as an evolutionary disadvantage but rather as a contribution to the evolvability of novel functions via exaptation.

## Introduction

Enzymes are remarkably specific catalysts and this characteristic led to the traditional view of “one enzyme, one substrate, one reaction”, which assumes an evolutionary preference for mono-functionality. However, it is clear now that enzymes can catalyze reactions other than those for which they evolved; see [1] and references therein. Prominent examples of multi-functional enzymes are glutathione S-transferases and cytochrome P450s, which can process several different substrates [1]. However, multi-functional enzymes may cause metabolic conflicts if they are involved in different, possibly independent, metabolic pathways [2]. Along these lines, multi-functionality seems to be of no immediate advantage for an organism, which argues against a positive selection of this trait. Presumably, neutral drift causes the broadening or narrowing of reaction specificity, see [1] and references therein; however it is unclear, whether multi-functionality is a short-term or a long-term trait.

Some evolutionary innovations originate non-adaptively as exaptations [3], *i*. *e*. as by-products of other, positively selected traits. These features were not built by natural selection for their current role. For example, feathers evolved for temperature regulation prior to their function in flight [3] and the light-refracting lens crystallins stem from enzymes [4]. *In silico* analyses suggest that exaptation is an important means of evolutionary innovation for metabolic systems [5]. The contribution of exaptation to evolutionary processes would be of even greater importance, if such traits existed over a long evolutionary time-span. In order to address this issue, we traced bi-functionality of a key metabolic enzyme over two billion years.

**Fig 1:**
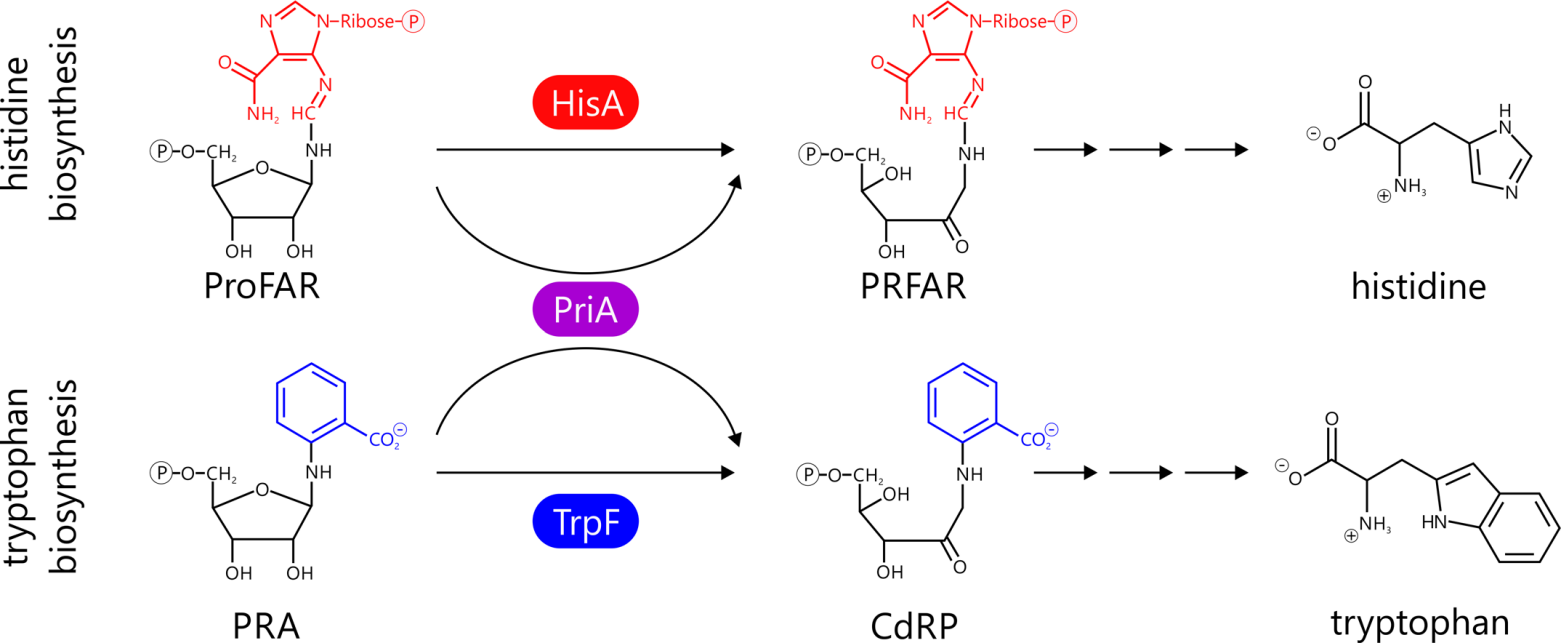
Isomerization of the aminoaldoses ProFAR and PRA to the aminoketoses PRFAR and CdRP. In most prokaryotes the two reactions are catalyzed by the enzymes HisA and TrpF, respectively. In Actinobacteria, however, the bi-functional PriA catalyzes both isomerizations.

Most microbial genomes harbor a *his*A and a *trp*F gene, which are located within the histidine and tryptophan operons, respectively. The gene products HisA and TrpF catalyze analogous isomerizations of the aminoaldoses ProFAR (Nʹ-[(5ʹ-phosphoribosyl)-formimino]-5-aminoimidazole-4-carboxamide-ribonucleotide) and PRA (N-(5ʹ-phosphoribosyl)anthranilate) into the aminoketoses PRFAR (Nʹ-[(5ʹ-phosphoribulosyl)-formimino]-5-aminoimidazole-4-carboxamide-ribonucleotide) and CdRP, respectively [6] (Fig 1). Most likely, genes for HisA and TrpF were present in the genome of the last universal common ancestor (LUCA) [7]; thus it can be expected that their modern successors process their specific substrates with high efficiency. The situation is different, however, in the Actinobacteria. Within this phylum, the *trp*F gene is missing in many genomes. As a substitute, the bi-functional isomerase PriA catalyzes both the HisA and the TrpF reactions [8] (Fig 1). PriA is a HisA homolog; the two enzymes are highly similar to each other with respect to sequence and structure [9, 10].

A detailed tracing of HisA bi-functionality required an analysis in two dimensions: A survey of PriA-like characteristics in modern HisA homologs and a retrospect of ancestors related to bacterial speciation. To begin with, we used *in silico* analyses and *in vitro* characterization of extant HisA enzymes and found that PriA-like bi-functionality is not strictly limited to Actinobacteria. Furthermore, we reconstructed *in silico* the sequences of the HisA/PriA ancestors of all Actinobacteria, all Proteobacteria, and all Bacteria, and tested the resulting precursor proteins for their ProFAR and PRA isomerase activities. Our results show that all three reconstructed ancestral enzymes are bi-functional *in vitro* and *in vivo*. Thus, our findings provide an example for an enzyme, whose bi-functionality pertained for two billion years of evolution, most likely without obvious, immediate benefit, except for exaptation.

## Results

### Occurrence and functional characterization of extant HisA and PriA enzymes

**Fig 2:**
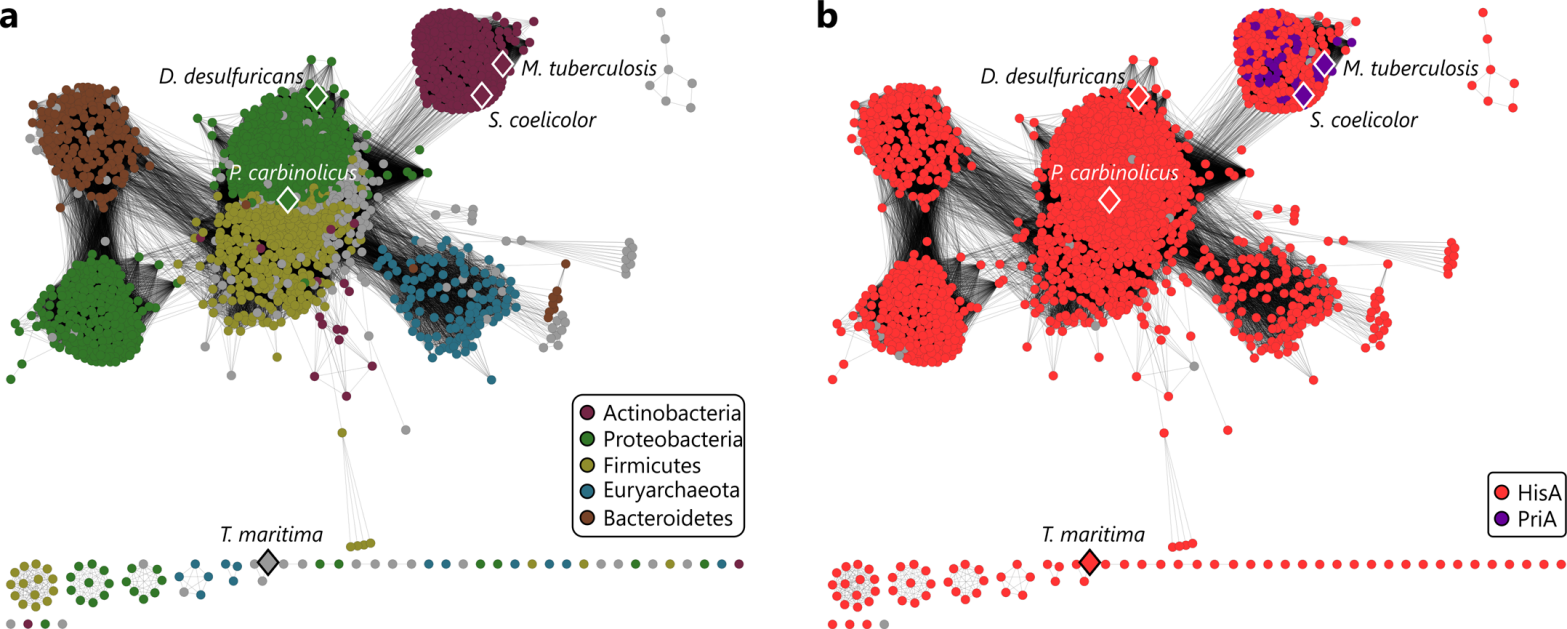
Sequence similarity network of the HisA/PriA superfamily. Nodes are colored by either the main five phyla contributing to this superfamily (A) or by annotation as HisA or PriA (B). The network was generated from all-by-all BLAST comparisons of 7428 HisA and PriA sequences. Each node represents a single sequence or a group of sequences with more than 95% identical residues; experimentally characterized HisA or PriA proteins are marked by diamonds. Each edge in the network represents a bi-directional BLAST hit with an E-value ≤ 1E−54 (corresponding to a median sequence identity of 44%). At this cutoff, the PriA cluster is clearly separated from, but still connected to the central HisA cluster. Lengths of edges are not meaningful except that sequences in tightly clustered groups are relatively more similar to each other than sequences with few connections.

The existence of the bi-functional PriA enzyme has originally been described for two actinobacterial species, namely *Streptomyces coelicolor* and *Mycobacterium tuberculosis* [8]. In order to determine the distribution of PriA-like enzymes within all bacterial phyla, we computed a sequence similarity network (SSN) of the HisA/PriA superfamily (Fig 2). In an SSN, nodes represent individual sequences and edges correspond to statistically significant similarities deduced from pairwise alignments, calculated by BLAST [11]. Our analysis showed that *his*A genes are present in all major phylogenetic groups (Fig 2A) and that the occurrence of annotated *pri*A genes is indeed restricted to the Actinobacteria (Fig 2B, top right cluster). The mean sequence identity in the Actinobacteria cluster is 52±9%; it can thus be assumed that all these sequences correspond to PriA enzymes.

**Fig 3:**
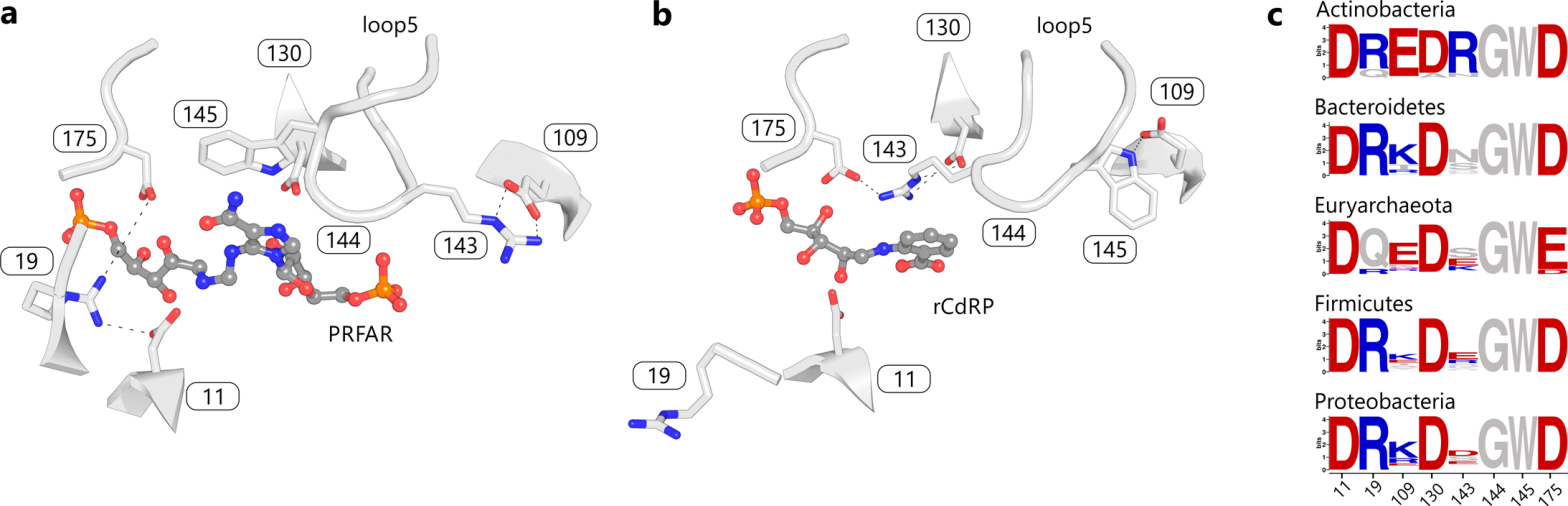
Two states of the PriA active site from *M*. *tuberculosis*. (a) Schematic view of the site in the HisA-state (bound product PRFAR, PDB ID 3zs4). (b) The same active site in the TrpF-state (bound product analogue reduced-CdRP, PDB ID 2y85). Residues of the active site are shown as stick models. Residue numbering is based on PDB ID 3zs4. (c) Sequence logos showing the conservation of the motif as deduced from SSN clusters of the HisA/PriA superfamily. Basic and acidic residues are colored blue and red, respectively.

The ability of PriA to catalyze both the HisA and the TrpF reaction requires that its active site can bind the two respective substrates in a productive conformation. As it is evident from the crystal structure of PriA from *M*. *tuberculosis* (mtPriA) [9], both substrates are bound in the same active site pocket (Fig 3). The most notable difference between the HisA state (Fig 3A) and the TrpF state (Fig 3B) is a twist of loop 5 and a concomitant swap of the localization of R143 and W145. This goes along with rearranged hydrogen bond networks at positions 19 and 109. Despite that, however, the same eight residues are involved in forming the active site in both states. We thus analyzed and compared their conservation in HisA and PriA sequences from the major SSN clusters. The actinobacterial PriA active site is characterized by a strong residue conservation resulting in the motif D-R-E-D-R-G-W-D (Fig 3C, Actinobacteria sequence logo). In contrast, the majority of HisA sequences deviate from the PriA-typical motif in 2-3 residues, mainly at positions 109 and 143 (Fig 3C, remaining sequence logos). Surprisingly, however, the PriA-typical motif is present in some HisA enzymes from Bacteroidetes (1 representative corresponding to 0.4% of all Bacteroidetes sequences), Euryarchaeota (6 / 5.1%), Firmicutes (25 / 8.9%), and Proteobacteria (43 / 4.9%). Moreover, the PriA-typical motif is also found in HisA from *Thermotoga maritima* (tmHisA), except that Lys is present at position 143 instead of the PriA-typical Arg.

In order to test if the presence of the PriA-typical active site sequence motif in HisA enzymes leads to TrpF activity, tmHisA and two HisA enzymes from Proteobacteria (*Pelobacter carbinolicus*, pcHisA; *Desulfovibrio desulfuricans*, ddHisA) were produced by heterologous gene expression in *Escherichia coli*. The recombinant proteins were purified and characterized by steady-state kinetics with respect to their ProFAR and PRA isomerization activities. Compared to PriA from *S*. *coelicolor* (scPriA) and *M*. *tuberculosis* (mtPriA), the catalytic efficiencies $k_{\text{cat}}/K_{\text{M}}^{\text{ProFAR}}$ of tmHisA, ddHisA, and pcHisA are about tenfold higher (Table 1, HisA reaction). They are comparable to the catalytic efficiency $k_{\text{cat}}/K_{\text{M}}^{\text{ProFAR}}$ of HisA from *Salmonella enterica* (seHisA), which is considered to be an archetypical representative of the HisA family [12]. Strikingly, tmHisA, ddHisA, and pcHisA also displayed TrpF-activity, something that has not been shown before. However, their catalytic efficiencies $k_{\text{cat}}/K_{\text{M}}^{\text{PRA}}$ are lower by about 10^5^–10^6^-fold compared to scPriA and mtPriA (Table 1, TrpF reaction).

**Table 1:** Steady-state kinetic parameters of extant PriA and HisA enzymes, and reconstructed HisA ancestors.

	HisA reaction			TrpF reaction		
Enzyme	*k*_cat_ [s^-1^]	$K_{\text{M}}^{\text{ProFar}}$ [μM]	$k_{\text{cat}}/K_{\text{M}}^{\text{ProFAR}}$ [M^-1^s^-1^]	*k*_cat_ [s^-1^]	$K_{\text{M}}^{\text{PRA}}$ [μM]	$k_{\text{cat}}/K_{\text{M}}^{\text{PRA}}$ [M^-1^s^-1^]
scPriA ^1^	0.9	28	3.2 · 10^4^	12	4	3.0 · 10^6^
mtPriA ^2^	0.23	19	1.2 · 10^4^	3.6	21	1.7 · 10^5^
tmHisA ^3^	1.0	5.6	1.8 · 10^5^	6.7 · 10^−3^	60	1.1 · 10^1^
ddHisA ^3^	1.3	2.8	4.6 · 10^5^	2.3 · 10^−3^	161	1.4 · 10^1^
pcHisA ^3^	0.4	1.8	2.2 · 10^5^	1.0 · 10^−3^	303	0.3 · 10^1^
seHisA ^4^	7.8	17.0	4.5 · 10^5^	n. d.	n. d.	n. d.
CA-Act-HisA ^5^	n. d.	n. d.	3.0 · 10^2^	1.0 · 10^−2^	3	3.3 · 10^3^
CA-Prot-HisA ^6^	0.05	0.3	1.8 · 10^5^	5.3 · 10^−4^	2.7	2.0 · 10^2^
CA-Bact-HisA ^6^	0.05	0.5	1.0 · 10^5^	2.3 · 10^−4^	3.2	0.7 · 10^2^

^1^ Data taken from [10].

^2^ Data taken from [9].

^3^ Unlike in previous work [13], tmHisA (as well as ddHisA and pcHisA) showed measurable albeit very low TrpF activity. Although the exact reasons for this discrepancy are unknown, it may be due to differences in enzyme purification and handling.

^4^ Data taken from [12]; n. d.: values were not determined.

^5^ n. d.: *k*_cat_ and $K_{\text{M}}^{\text{ProFAR}}$ could not be determined individually; $k_{\text{cat}}/K_{\text{M}}^{\text{ProFAR}}$ was deduced from the linear part of the saturation curve.

^6^ The *k*_cat_ and $K_{\text{M}}^{\text{ProFAR}}$ values were determined by analyzing entire transition curves with the integrated Michaelis-Menten equation.

^3,5,6^ The standard errors for *k*_cat_ and *K*_*M*_ were between 5% and 40%.

*In vivo* complementation experiments showed that tmHisA, ddHisA, and pcHisA were able to rescue the growth deficiency of an *E*. *coli* Δ*his*A strain. Moreover, despite their weak *in vitro* TrpF activity, they were also able to complement a Δ*trp*F strain (Table 2). The enzymes were further able to complement a Δ*his*AΔ*trp*F double deletion strain (Table 2), whereby the time required for complementation is clearly limited by their TrpF activity.

**Table 2:** *In vivo* complementation of auxotrophic *E*. *coli* strains by PriA, HisA, HisA ancestors, and TrpF.

	complementation time in hours		
	Δ*his*A strain	Δ*trp*F strain	Δ*his*AΔ*trp*F strain
scPriA	22	22	23
tmHisA	16	114	144
ddHisA	16	153	181
pcHisA	15	70	63
CA-Act-HisA	48	23	47
CA-Prot-HisA	16	33	28
CA-Bact-HisA	16	45	39
tmTrpF	no growth	24	no growth

For all experiments, the mean time is given after which visible colonies appeared on minimal medium agar plates. All experiments were repeated independently at least three times. A growth time of 16 hours indicates that colonies appeared over night. Growth times below 120 hours could be reproduced with a maximum error of 25%, growth times above 120 hours with a maximum error of 40%. “No growth” indicates that no colonies were observed after 14 days. A negative control with empty pTNA plasmid did not lead to growth within 14 days, either.

### Reconstruction of ancient sequences

We next asked whether the bi-functionality of HisA is an ancient feature that has been conserved in certain extant enzymes. To this end, we computationally reconstructed three HisA precursors as described in the following. It has been shown that concatenating related sequences increases the strength of the phylogenetic signal available for tree construction [14]. Thus, we concatenated species-wise HisA with HisH and HisF sequences. The respective genes were most likely part of the LUCA genome [7] and have remained elements of the histidine operon since then. Bacterial and archaeal genomes were scanned for the occurrence of *his*A genes, and species were selected for which *his*A, *his*F, and *his*H were gene neighbors. We picked sequences from Euryarchaeota (5 species), Crenarchaeota (20), Bacteroidetes (8), Firmicutes (11), Spirochaetes (5), and the α-, β-, γ-, and δ-Proteobacteria (21, 5, 1, 5). Moreover, we added 22 actinobacterial sequence sets, by selecting genes whose products contain the above mentioned PriA active site sequence motif.

The resulting *MSA*_*HisFAH*_ comprised 103 concatenations (species names listed in S1 Table). After preprocessing this input, a phylogenetic tree was determined and assessed by means of PhyloBayes v3.3 [15]. Four independent MCMC samplings of length 50,000 were computed using pb and compared to ensure convergence. Several parameters confirmed the validity of our approach: Convergence and mixing were checked by means of the discrepancy index maxdiff; for the pairwise comparison of all chains, the maxdiff value was at most 0.06. The effective size was at least 100, as determined by means of tracecomp. A consensus tree was deduced from the concatenation of these four chains (S1 Fig). The posterior probability of edges interlinking ancestors of phyla or classes was at least 0.87, which testifies to the high quality of the tree.

**Fig 4:**
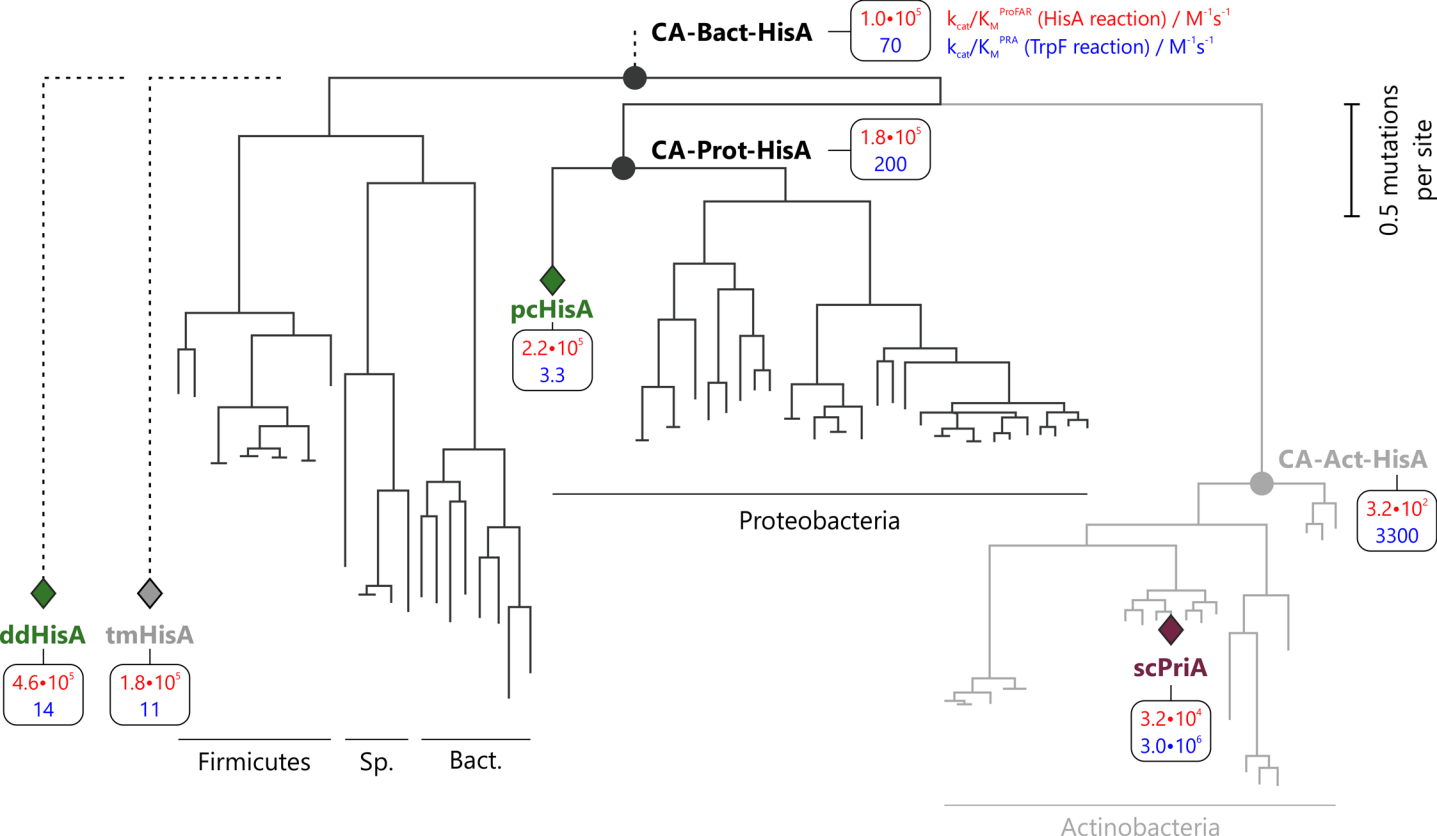
Phylogenetic tree depicting the position of extant HisA and PriA enzymes (diamonds) and their relationship to the reconstructed ancestral HisA enzymes (circles). The topology of the tree was inferred from the phylogenetic trees used for sequence reconstruction (S1 and S2 Figs). CA-Act-HisA, CA-Prot-HisA, and CA-Bact-HisA are the predecessor of HisA enzymes from Actinobacteria, Proteobacteria and Bacteria, respectively. Note that actinobacterial sequences were omitted for reconstruction of CA-Prot-HisA and CA-Bact-HisA (indicated by grey shading of the Actinobacteria branch). ddHisA and tmHisA were not used for sequence reconstruction and are only listed because they were characterized experimentally. The vertical bar indicates the branch length that corresponds to 0.5 mutations per site. The catalytic efficiencies *k*_cat_/*K*_M_ of the enzymes for processing ProFAR and PRA are given in red and blue, respectively. Abbreviations: sc, *S*. *coelicolor*; dd, *D*. *desulfuricans*; pp, *P*. *carbinolicus*; tm, *T*. *maritima*; Sp., Spirochaetes; Bact., Bacteroidetes.

This tree and the corresponding *MSA*_*HisFAH*_ were used to deduce a predecessor of the actinobacterial enzymes (CA-Act-HisA) by means of FASTML [16]. In order to exclude any effect of the 22 actinobacterial sequences (and especially their active site motif) on the reconstruction of more ancient predecessors of HisA, these sequences were removed from *MSA*_*HisFAH*_. The resulting *MSA*_*HisFAH-Act*_, which contained the remaining 81 non-actinobacterial sequences, was used to calculate a second tree (S2 Fig). Applying FASTML, the sequences of the common ancestors of Proteobacteria (CA-Prot-HisA) and of Bacteria (CA-Bact-HisA) were determined. A schematic representation of the two trees is given in Fig 4. The archaeal sequences served as an outgroup in both reconstructions.

### Experimental assessment of HisA precursors

The genes coding for the three precursors were synthesized and heterologously expressed in *E*. *coli*. The recombinant proteins were soluble and stable, and could be purified. Steady-state kinetic analysis yielded $k_{\text{cat}}/K_{\text{M}}^{\text{ProFAR}}$ values in the order of 10^2^–10^5^ M^-1^s^-1^ for the HisA reaction, and $k_{\text{cat}}/K_{\text{M}}^{\text{PRA}}$ values in the order of 10^2^–10^3^ M^-1^s^-1^ for the TrpF reaction (Table 1). Compared to scPriA and mtPriA, the catalytic efficiency of the ancestral proteins for the TrpF reaction is therefore only two to three orders of magnitude lower. For all three proteins this is the result of a lower *k*_cat_ value; the $K_{\text{M}}^{\text{PRA}}$ is practically identical to that of scPriA. Furthermore, all three precursors were able to complement the growth deficiencies of Δ*his*A and Δ*trp*F strains (Table 2). The time required for *in vivo* complementation agrees well with *k*_cat_/*K*_M_ values determined from *in vitro* measurements. For example, CA-Bact-HisA and CA-Prot-HisA have the highest $k_{\text{cat}}/K_{\text{M}}^{\text{ProFAR}}$ values and required the least time to complement the Δ*his*A strain. CA-Act-HisA has the highest $k_{\text{cat}}/K_{\text{M}}^{\text{PRA}}$ value and required the least time to complement the Δ*trp*F strain. All three HisA-ancestors were further able to complement the growth deficiency of a Δ*his*AΔ*trp*F double deletion strain (Table 2). The observed complementation times agree well with those determined from the single deletion strains. The complementation by CA-Act-HisA is limited by its ability to compensate for the missing HisA reaction, whereas complementation by CA-Prot-HisA and CA-Bact-HisA is limited by their ability to catalyze the missing TrpF reaction.

The active site sequence motif of CA-Act-HisA is identical to that of modern PriA enzymes. The motifs of CA-Prot-HisA and CA-Bact-HisA match in six of the eight residues. Non-matching is position 109, which contains a Lys instead of a Glu. At the second non-matching position 143, both precursors contain a Lys instead of an Arg. It is therefore plausible to assume that a basic residue at position 143 is crucial for bi-functionality. In contrast, the recently published SGG sequence motif of PriA [17] seems not to be required for bi-functionality. Only the immediate actinobacterial precursor CA-Act-HisA contains the SGG-motif whereas both other precursors displayed significant bi-functionality albeit containing a GGG-motif.

## Discussion

In contrast to previous results [18], the reconstructed CA-Prot-HisA and CA-Bact-HisA are to our knowledge the first examples of ancestral metabolic enzymes from approximately 2.5 to 2.0 billion years ago [19] that were shown to be bi-functional. This trait is even more interesting when one considers that only extant HisA sequences but no extant PriA sequences were selected to reconstruct the CA-Prot-HisA and CA-Bact-HisA predecessors.

Strikingly, we also detected bi-functionality in the modern tmHisA, pcHisA, and ddHisA and thus provide the first examples of HisA/TrpF bi-functionality in extant HisA enzymes. It is worth noting that these three species all contain a *trp*F gene, which suggests that no selective pressure exists for these species to maintain the bi-functionality in HisA. Moreover, the *in vivo* complementation experiments show that tmTrpF is functional and is able to rescue an *E*. *coli* Δ*trp*F strain (Table 2). Also, the bi-functionality of these modern HisA enzymes does not force their hosts to face functional trade-offs because $K_{\text{M}}^{\text{PRA}}$ values are 10- to 170-fold higher than $K_{\text{M}}^{\text{ProFAR}}$ values. Thus the obligate HisA activity of these enzymes is most likely not impaired by the binding of PRA or CdRP. Moreover, the catalytic efficiencies $k_{\text{cat}}/K_{\text{M}}^{\text{PRA}}$ are in a physiologically irrelevant range below 14 M^-1^s^-1^ thus making TrpF side-activity tolerable. Along these lines, the CA-Bact-HisA predecessor evolved most likely in a similar way such that the remaining TrpF side-activity was physiologically not harmful.

Our results do not allow us to decide whether all modern HisA enzymes are bi-functional: We have performed *in vivo* complementation experiments with four additional HisA enzymes from Bacteroidetes, Firmicutes, Proteobacteria, and Euryarchaeota lacking the PriA-typical sequence motif. These enzymes were unable to rescue Δ*trp*F or Δ*his*AΔ*trp*F deletion strains within eight days. Nevertheless, extremely slow growing colonies were observed occasionally. This growth may be due to residual TrpF activity of inherent *E*. *coli* enzymes like PurF [20] and may therefore indicate the existence of additional routes of exaptation. The active site motifs (Fig 3) suggest that bi-functionality is determined by Glu 109 and Arg 143. HisA homologs that retained bi-functionality have conserved the PriA typical residues at these two positions, despite a relatively low overall sequence identity. As this bi-functionality seems to be neither beneficial nor harmful for an organism, we assume that its presence is simply a matter of historical contingency. This conclusion is in agreement with the finding that a few mutations acquired in not more than several thousand generations were sufficient to transform a bi-functional HisA variant from *S*. *enterica* into a specialized HisA enzyme lacking TrpF activity or *vice versa* [21]. Along these lines, the bi-functional PriA became a mono-functional HisA enzyme in the Corynebacteria, a distinct genus within the Actinobacteria. This re-narrowing of substrate specificity in the so-called subHisA occurred after the horizontal acquisition of a whole pathway tryptophan operon (including a *trp*F gene) from a member of the γ-Proteobacteria [22]. Again, this transition from a bi-functional PriA to a mono-functional HisA enzyme required only subtle sequence alterations [17]. Noteworthy is a change from Arg 143 to an Asn, which supports the important role of Arg 143 for bi-functionality. Again, mono-functionality of HisA is easily accessible, if under evolutionary constraints. For Corynebacteria, this evolutionary pressure is most likely due to a metabolic conflict between histidine and tryptophan biosynthesis.

This bi-functionality provided a means for compensating the loss of the *trp*F gene within the Actinobacteria. Importantly, such exaptations are not rare: A screening of 104 single-gene knockout strains made clear that approximately 20% of these auxotrophs were rescued by the overexpression of at least one noncognate *E*. *coli* gene [23]. Thus, the functional diversity of gene products contributes to metabolic robustness and evolvability. These evolutionary advantages are further increased, if a bi-functionality that confers no cost or benefit to organismal fitness, can be conserved throughout long evolutionary time-spans. The characteristics of ancient and extant HisA and PriA enzymes confirm that this is feasible, even for enzymes of the primary metabolism.

## Materials and Methods

### Generation of sequence similarity networks

The SSN of the HisA/PriA-superfamily (7824 sequences, IPR023016 from InterPro release 47.0 [24]) was created using standard methods [25] provided by the Enzyme Function Initiative [26]. In order to eliminate sequence fragments, the length of the sequences that were included in the all-by-all BLAST comparison was restricted to 230–260 amino acids. From the remaining 7428 sequences, a representative network with an E-value cut-off of 1E-54 was generated in which sequences that share >95% identity were grouped into single nodes by CD-HIT [27]. Detailed phylogenetic information (superkingdom, phylum, class, order, family, genus) was added for each node using a modified version of Key2Ann [28]. Networks were visualized with the organic y-files layout in Cytoscape 3.2.0 [29, 30]. Phylum-specific sequence sets were compiled from the SSN and used to compute sequence logos of the active site residues, essentially as described [31].

### Reconstruction of ancestral sequences

BLAST [11] and the nr database of the NCBI were used to search for the sequences of HisA homologs in completely sequenced genomes. Species where chosen, where *his*A and the *his*F and *his*H genes were neighbors in the genome; the respective sequences were concatenated. We selected species from the archeal phyla Euryarchaeota and Crenarchaeota, and from the bacterial phyla Bacteroidetes, Firmicutes, Spirochaetes, Actinobacteria, and Proteobacteria. A multiple sequence alignment (MSA) was deduced by means of MAFFT [32]. Positions containing more than 50% gaps were removed by using GBlocks [33]. The resulting MSA contained 430 meaningful positions. The program pb (version 3.3 of PhyloBayes, [15]) with options–cat–gtr was used to compute in four independent Monte Carlo Markov Chains (MCMC) 50 000 samples each. The options–cat–gtr induce an infinite mixture model, whose components differ by their equilibrium frequencies. The quality of mixing was assessed by computing the discrepancy index (maxdiff) by means of bpcomp and the minimum effective size with tracecomp. A consensus tree was determined by means of readpb, the burnin was 5000.

An MSA and a rooted tree determined as described were the input for FASTML [16]. The JTT substitution model and the maximum likelihood method were used for indel reconstruction. As a representative predecessor, we chose the most likely sequence related to the respective node of the phylogenetic tree. Nucleotide and amino acid sequences of synthesized genes for ancestral proteins are given in S2 Table.

### Site directed mutagenesis and cloning

A list of all oligonucleotides used for cloning and site-directed mutagenesis is provided in S3 Table. The sc*pri*A gene from *S*. *coelicolor*, which served as a positive control in the *in vivo* complementation assays, was amplified from scPriA-pTYB4 (a gift of Dr. Matthias Wilmanns) by standard PCR, using the oligonucleotides 5ʹsc*pri*A_*Sph*I/3ʹsc*pri*A_ Stop_*Hind*III, and cloned into the pTNA vector [6] via the introduced restriction sites for *Sph*I and *Hind*III. The tm*trpF* gene from *T*. *maritima*, which served as a negative control in the *in vivo* complementation assays, was available in a pTNA vector from previous work [34].

The *his*A gene from *T*. *maritima* (tm*his*A) was amplified using the template pDS56/RBSII_*his*A [35] with the oligonucleotides 5ʹtm*his*A_*Nde*I/3ʹtm*his*A_*Not*I (pET21a) and 5ʹtm*his*A_*Sph*I/3ʹtm*his*A_Stopp_*Hin*dIII (pTNA) and subsequently cloned into pET21a (Stratagene) and pTNA vectors using the respective terminal restriction sites. The genomic DNA of *D*. *desulfuricans ssp*. *Desulfuricans* and *P*. *carbinolicus* were ordered from DSMZ (DSM2380 and DSM6949, respectively). The respective *his*A genes (dd*his*A and pc*his*A) were amplified in a standard PCR using the oligonucleotides 5ʹdd*his*A_*Nde*I/3ʹdd*his*A_*Xho*I and 5ʹpc*his*A_*Nde*I/3ʹpc*his*A_*Xho*I, respectively, and subsequently cloned into the pET24a vector (Stratagene) via the introduced restriction sites for *Nde*I and *Xho*I. For *in vivo* complementation assays both *his*A genes were cloned into the pTNA vector via the restriction sites for *Sph*I and *Hind*III. To this end, pc*his*A was amplified with the oligonucleotides 5ʹpc*his*A_*Sph*I and 3ʹpc*his*A_Stopp_*Hind*III, whereas in the case of dd*his*A an overlap extension PCR [36] was necessary to remove an intrinsic *Sph*I restriction site. This reaction was performed with the oligonucleotides 5ʹdd*his*A_*Sph*I, 3ʹdd*his*A_C516T, 5ʹdd*his*A_C516T, and 3ʹdd*his*A_Stopp_*Hind*III.

The genes coding for the reconstructed ancestors were optimized for their expression in *E*. *coli*, synthesized (LifeTechnologies), and cloned into the pTNA and pET24a vectors using the terminal restriction sites for *Sph*I and *Hind*III. In order to render pET24a compatible for cloning with *Sph*I, two QuikChange mutagenesis steps were performed: the *Nde*I restriction site of pET24a was replaced by a *Sph*I restriction site using the oligonucleotides 5ʹpET24a_*Nde*I_to_*Sph*I and 3ʹpET24a_*Nde*I_to_*Sph*I, whereas a *Sph*I restriction site remote from the multiple cloning site was removed using the oligonucleotides 5ʹpET24a_A536T and 3ʹpET24a_A536T. All gene constructs were entirely sequenced to exclude inadvertent mutations.

### Heterologous expression and purification of recombinant proteins

Gene expression, harvesting of cells, and cell lysis were performed essentially as described [18]. The genes pc*his*A and dd*his*A were expressed in *E*. *coli* T7 Express cells (New England Biolabs) containing the pRARE helper plasmid [34]. The gene tm*his*A was expressed in *E*. *coli* BL21-CodonPlus-(DE3)-RIPL cells (Agilent Technologies). The genes for the reconstructed proteins were expressed in *E*. *coli* BL21-Gold (DE3) cells (Agilent Technologies). For purification of tmHisA, heat denaturation (70°C, 15 min) was performed to remove most of the host proteins. Soluble cell extracts were loaded onto a HisTrapFF crude column (5 mL; GE Healthcare), which had been equilibrated with 50 mM potassium phosphate, pH 7.5, 300 mM sodium chloride, and 10 mM imidazole. After washing with equilibration buffer, the bound protein was eluted by applying a linear gradient of 10–375 mM imidazole. Subsequently, fractions with pure protein were pooled and dialyzed twice against 50 mM Tris·HCl, pH 7.5. Before dialyzing the reconstructed proteins CA-Bact-HisA, CA-Prot-HisA, and CA-Act-HisA in the same manner, fractions containing the respective protein were loaded onto a Superdex75 column (HiLoad 26/60, 320 mL, GE Healthcare) operated with 50 mM Tris·HCl, pH 7.5, and 50 mM sodium chloride at 4°C. In all cases, at least 1 mg protein was obtained per liter of culture. All proteins were more than 95% pure, as judged by SDS-PAGE.

### Steady-state enzyme kinetics

The HisA reaction was measured spectrophotometrically at 300 nm and 25°C as described [6]. The TrpF reaction was followed at 25°C by a fluorimetric assay (excitation at 350 nm, emission at 400 nm) [37]. The substrate PRA was generated *in situ* from anthranilate and phosphoribosylpyrophosphate (PRPP) using 1 μM yeast anthranilate phosphoribosyl transferase. To assure a constant concentration of the unstable PRA during the individual TrpF activity measurements, a 30-fold molar excess of PRPP over anthranilate was used. The *k*_cat_ and *K*_M_ values for both reactions were determined by fitting the hyperbolic saturation curves with the Michaelis-Menten equation. For unknown reasons, the CA-Prot-HisA and CA-Bact-HisA proteins exhibited a strong hysteresis, both in the HisA and TrpF reaction. Therefore, entire progress curves were recorded starting with as many as five different initial substrate concentrations. The curves were analyzed with COSY [38] using the integrated Michaelis-Menten equation for progress curves of the HisA reaction and a Michaelis-Menten equation that includes product inhibition for progress curves of the TrpF reaction.

### *E*. *coli* knockout strains

The *E*. *coli* Δ*his*A strain was generated according to a classical protocol [39]. In brief, an ampicillin resistance gene was integrated into an *E*. *coli* DY329 helper strain to replace the genomic *his*A gene with the aid of this strain’s genetically encoded bacteriophage λ Red recombination system [40]. The resistance gene was then transferred to *E*. *coli* BW25113 via P1 phage transduction and replaced the genomic *his*A gene. The complete deletion of the *his*A gene was verified by sequencing. The *E*. *coli* Δ*his*AΔ*trp*F double deletion strain was generated from the Δ*his*A strain in the same manner, with the genomic *trp*F gene being replaced by a chloramphenicol resistance gene. The *E*. *coli* Δ*trp*F single deletion strain (*E*. *coli* JMB9r-m+Δ*trp*F) was available from previous work [41].

### *In vivo* complementation assays

Complementation assays with pTNA_sc*pri*A, pTNA_tm*his*A, pTNA_tm*trp*F, pTNA_dd*his*A, and pTNA_pc*his*A, as well as with the pTNA constructs of the reconstructed ancestors CA-Act-HisA, CA-Prot-HisA, and CA-Bact-HisA were performed on M9 minimal medium agar plates. An identical experimental procedure was followed in all cases: First, the respective plasmid was used to transform either chemical competent Δ*his*A, Δ*trp*F, or Δ*his*AΔ*trp*F *E*. *coli* cells. Next, single colonies were picked in order to inoculate 5 mL of LB medium supplemented with 150 μg/mL ampicillin only (Δ*his*A cells) or with 150 μg/mL ampicillin and 30 μg/mL chloramphenicol (Δ*trp*F and Δ*his*AΔ*trp*F cells). After incubation at 37°C overnight, 5 mL of LB medium containing the respective resistance markers were inoculated (optical density of 0.1 at 600 nm) and incubated at 37°C until an optical density of about 1 at 600 nm was reached (corresponding to approximately 10^8^ cells). Subsequently, the cells in 1 mL suspension were collected by centrifugation (4°C, 4000 *g*, 10 min) and washed three times with 1% NaCl. Finally, 1:10^5^ and 1:10^4^ dilutions were streaked out on M9 minimal medium agar plates containing 150 μg/mL ampicillin and incubated at 37°C.

## Supporting Information

S1 Fig Phylogenetic tree based on 103 HisA sequences. Each sequence consists of the concatenated sequences of a HisA, a HisF, and a HisH protein. The tree was determined using pb, which is part of the PhyloBayes package. Posteriori probabilities are given for the splits; the length of the bar at the top corresponds to 0.5 mutations per site. Names encode the phylogenetic lineage of the species, see S1 Table. The node that corresponds to the reconstructed common ancestor of Actinobacteria (CA-Act-HisA) is marked with a filled circle.
(PDF)

S2 Fig Phylogenetic tree based on 81 HisA sequences. Each sequence set consists of the concatenated sequences of a HisA, a HisF, and a HisH protein. The tree was determined using pb, which is part of the PhyloBayes package. Posteriori probabilities are given for the splits; the length of the bar at the top corresponds to 0.5 mutations per site. Names encode the phylogenetic lineage of the species, see S1 Table. The nodes that correspond to the reconstructed common ancestor of Proteobacteria (CA-Prot-HisA) and Bacteria (CA-Bact-HisA) are marked with a filled circle.
(PDF)

S1 Table Species names and their abbreviations. The data set for the determination of a phylogenetic tree consisted of concatenated sequences of one HisA, one HisF, and one HisH, originating from the species listed below. For each phylum, the number of sequences is given in brackets. In the list, each species name is followed by the abbreviation (in brackets) used to label leaves of phylogenetic trees. The first symbol of the abbreviation indicates the superkingdom, the next four groups of two characters each give phylum, class, order, family, and the last three characters indicate the species name. Additional numbers were added by the algorithm used to create the abbreviations [28] but have no meaning in this context.
(PDF)

S2 Table Nucleotide and amino acid sequences of synthesized genes for ancestral proteins. (PDF)

S3 Table List of oligonucleotides used for cloning and site-directed mutagenesis. (PDF)
