# Supplementary figures and images for: Long-Term Persistence of Bi-functionality Contributes to the Robustness of Microbial Life through Exaptation
Source: PLoS Genet. 2016 Jan 29;12(1):e1005836. doi: 10.1371/journal.pgen.1005836 (PMC4732765; doi:10.1371/journal.pgen.1005836)

Supplementary Figure 1

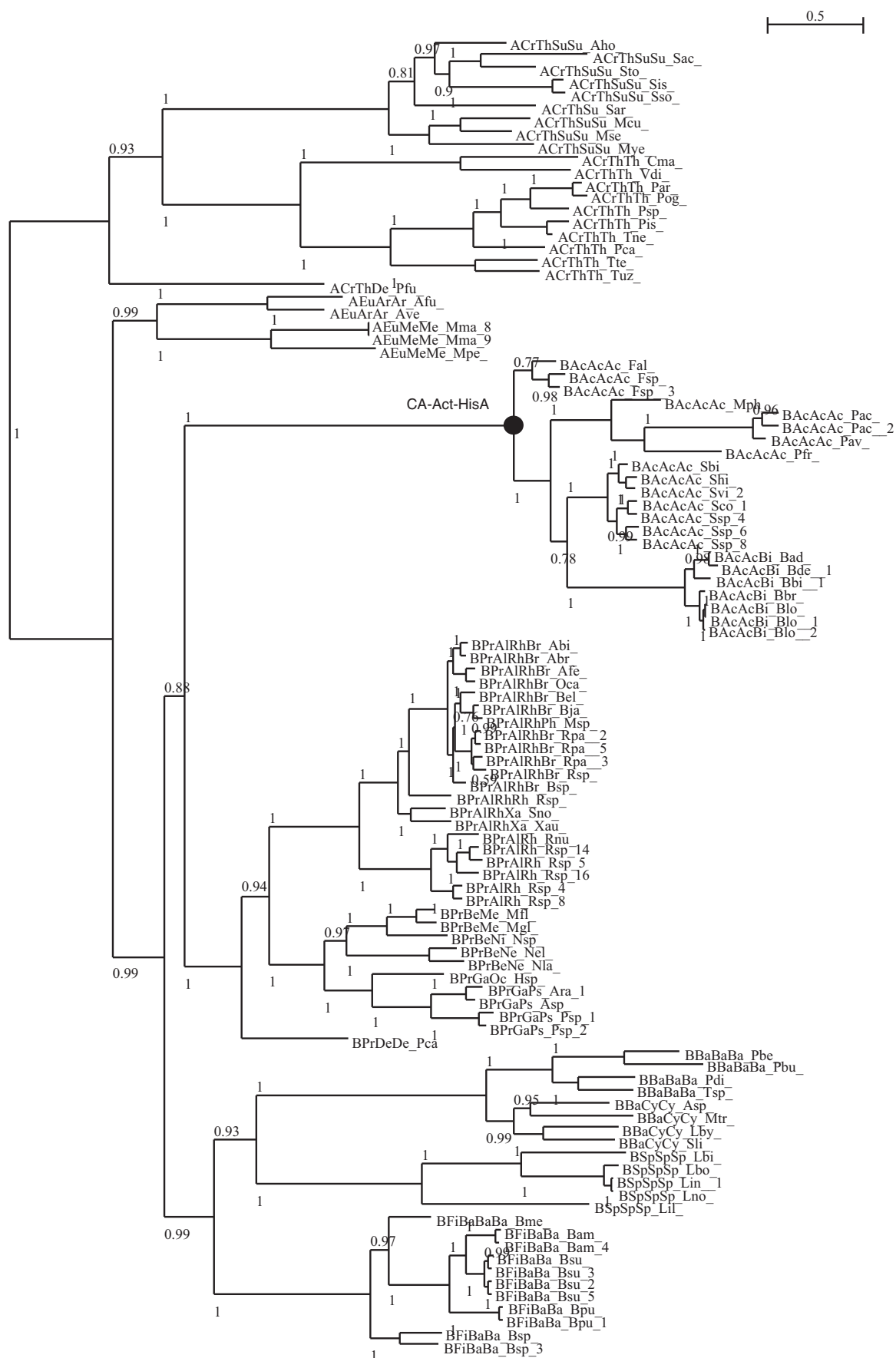

Supplement: S1 Fig — Each sequence consists of the concatenated sequences of a HisA, a HisF, and a HisH protein. The tree was determined using pb, which is part of the PhyloBayes package. Posteriori probabilities are given for the splits; the length of the bar at the top corresponds to 0.5 mutations per site. Names encode the phylogenetic lineage of the species, see S1 Table. The node that corresponds to the reconstructed common ancestor of Actinobacteria (CA-Act-HisA) is marked with a filled circle. (PDF) [file pgen.1005836.s001.pdf]

Supplementary Figure 2

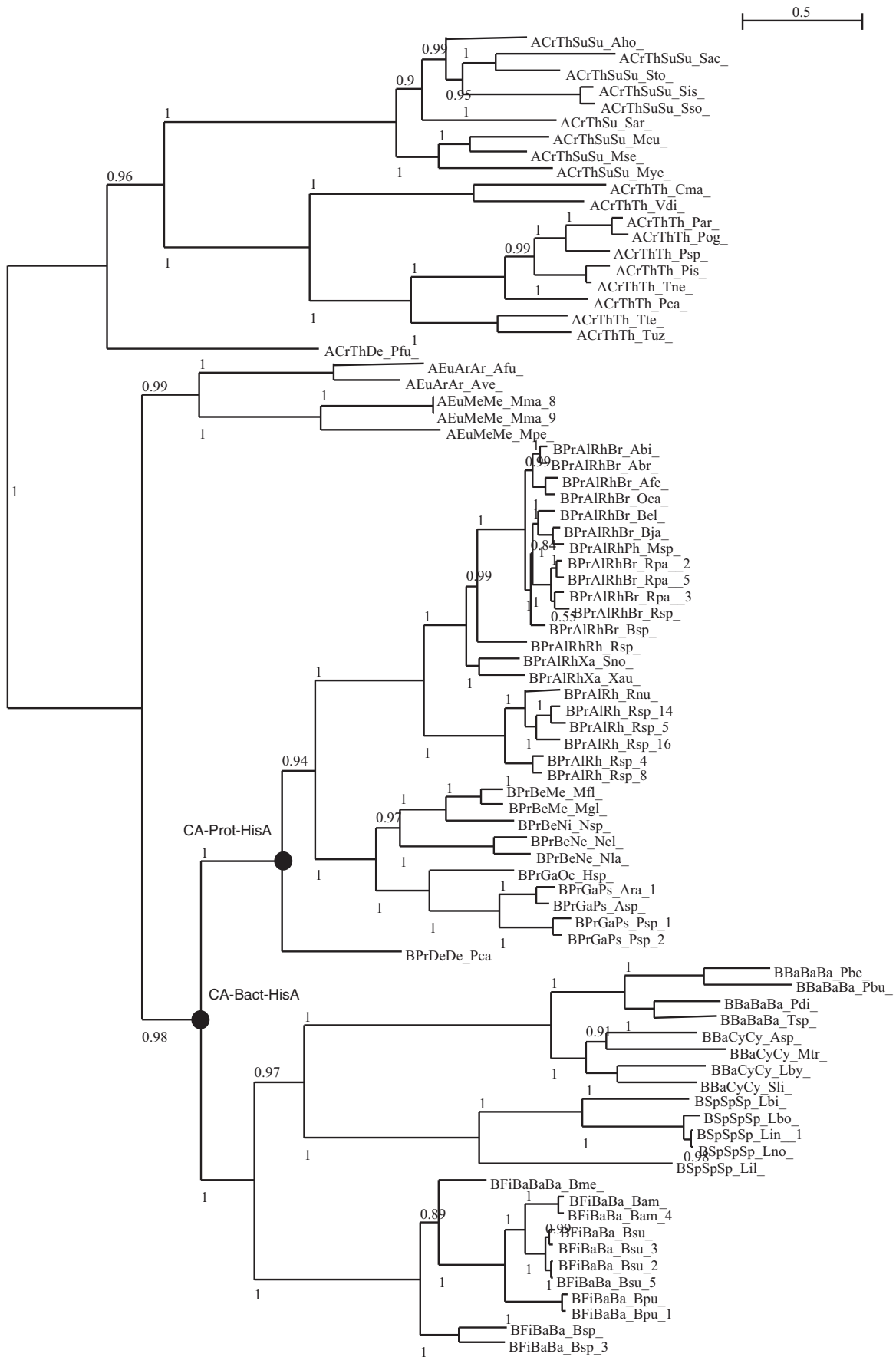

Supplement: S2 Fig — Each sequence set consists of the concatenated sequences of a HisA, a HisF, and a HisH protein. The tree was determined using pb, which is part of the PhyloBayes package. Posteriori probabilities are given for the splits; the length of the bar at the top corresponds to 0.5 mutations per site. Names encode the phylogenetic lineage of the species, see S1 Table. The nodes that correspond to the reconstructed common ancestor of Proteobacteria (CA-Prot-HisA) and Bacteria (CA-Bact-HisA) are marked with a filled circle. (PDF) [file pgen.1005836.s002.pdf]
