# Supplementary material for: Long-Term Persistence of Bi-functionality Contributes to the Robustness of Microbial Life through Exaptation
Source: PLoS Genet. 2016 Jan 29;12(1):e1005836. doi: 10.1371/journal.pgen.1005836 (PMC4732765; doi:10.1371/journal.pgen.1005836)
Supplement: S1 Table — The data set for the determination of a phylogenetic tree consisted of concatenated sequences of one HisA, one HisF, and one HisH, originating from the species listed below. For each phylum, the number of sequences is given in brackets. In the list, each species name is followed by the abbreviation (in brackets) used to label leaves of phylogenetic trees. The first symbol of the abbreviation indicates the superkingdom, the next four groups of two characters each give phylum, class, order, family, and the last three characters indicate the species name. Additional numbers were added by the algorithm used to create the abbreviations [28] but have no meaning in this context. (PDF) [file pgen.1005836.s003.pdf]

S1 Table: Species names and their abbreviations

The data set for the determination of a phylogenetic tree consisted of concatenated sequences of one HisA, one HisF, and one HisH, originating from the species listed below. For each phylum, the number of sequences is given in brackets. In the list, each species name is followed by the abbreviation (in brackets) used to label leaves of phylogenetic trees. The first symbol of the abbreviation indicates the superkingdom, the next four groups of two characters each give phylum, class, order, family, and the last three characters indicate the species name. Additional numbers were added by the algorithm used to create the abbreviations [1] but have no meaning in this context.

Crenarchaeota (20): *Vulcanisaeta distributa* (ACrThTh_Vdi), *Pyrolobus fumarii* (ACrThDe_Pfu), *Acidianus hospitalis* (ACrThSuSu_Aho), *Metallosphaera cuprina* (ACrThSuSu_Mcu), *Metallosphaera sedula* (ACrThSuSu_Mse), *Metallosphaera yellowstonensis* (ACrThSuSu_Mye), *Sulfolobus acidocaldarius* (ACrThSuSu_Sac), *Sulfolobus islandicus* (ACrThSuSu_Sis), *Sulfolobus solfataricus* (ACrThSuSu_Sso), *Sulfolobus tokodaii* (ACrThSuSu_Sto), *Sulfolobales archaeon Acd1* (ACrThSu_Sar), *Caldivirga maquilingensis* (ACrThTh_Cma), *Pyrobaculum arsenaticum* (ACrThTh_Par), *Pyrobaculum calidifontis* (ACrThTh_Pca), *Pyrobaculum islandicum* (ACrThTh_Pis), *Pyrobaculum oguniense* (ACrThTh_Pog), *Pyrobaculum sp. 1860* (ACrThTh_Psp), *Thermoproteus neutrophilum* (ACrThTh_Tne), *Thermoproteus tenax* (ACrThTh_Tte), *Thermoproteus uzoniensis* (ACrThTh_Tuz)

Euryarchaeota (5): *Archaeoglobus veneficus* (AEuArAr_Ave), *Methanoculleus marisnigri* (AEuMeMe_Mma_8), *Methanoculleus marisnigri* (AEuMeMe_Mma_9), *Methanoplanus petrolearius* (AEuMeMe_Mpe), *Archaeoglobus fulgidus* (AEuArAr_Afu)

Bacterioidetes (8): *Prevotella buccae* (BBaBaBa_Pbu), Parabacteroides distasonis (BBaBaBa_Pdi), *Tannerella sp. 6_1_58FAA_CT1* (BBaBaBa_Tsp), *Algoriphagus machipongonensis* (BBaCyCy_Asp), *Leadbetterella byssophila* (BBaCyCy_Lby), *Marivirga tractuosa* (BBaCyCy_Mtr), *Spirosoma linguale* (BBaCyCy_Sli), *Prevotella bergensis* (BBaBaBa_Pbe)

Firmicutes (11): *Bacillus amyloliquefaciens* (BFiBaBa_Bam), *Bacillus amyloliquefaciens* (BFiBaBa_Bam_4), *Bacillus pumilus* (BFiBaBa_Bpu), *Bacillus pumilus* (BFiBaBa_Bpu_1), *Bacillus* sp. NRRL B-14911 (BFiBaBa_Bsp), *Bacillus sp. 1NLA3E* (BFiBaBa_Bsp_3), *Bacillus subtilis* (BFiBaBa_Bsu), *Bacillus subtilis* (BFiBaBa_Bsu_2), *Bacillus subtilis* (BFiBaBa_Bsu_3), *Bacillus subtilis* (BFiBaBa_Bsu_5), *Bacillus* megaterium (BFiBaBaBa_Bme)

Spirochaetes (5): *Leptospira biflexa* (BSpSpSp_Lbi), *Leptospira* borgpetersenii (BSpSpSp_Lbo), *Leptonema illini* (BSpSpSp_Lil), *Leptospira* (BSpSpSp_Lin__1), *Leptospira noguchii* (BSpSpSp_Lno)

Proteobacteria (32): *Afipia birgiae* (BPrAlRhBr_Abi), *Afipia bromae* (BPrAlRhBr_Abr), *Afipia felis* (BPrAlRhBr_Afe), *Bradyrhizobium elkanii* (BPrAlRhBr_Bel), *Bradyrhizobium japonicum* (BPrAlRhBr_Bja), *Bradyrhizobium sp. BTAi1* (BPrAlRhBr_Bsp), *Oligotropha carboxidovorans* (BPrAlRhBr_Oca), *Rhodopseudomonas palustris* (BPrAlRhBr_Rpa__*2), Rhodopseudomonas palustris* (BPrAlRhBr_Rpa__*3), Rhodopseudomonas palustris* (BPrAlRhBr_Rpa__*5), Rhodopseudomonas sp. B29* (BPrAlRhBr_Rsp), *Bradyrhizobium sp. WSM4349* (BPrAlRhPh_Msp), *Rhodovulum sp. PH10* (BPrAlRhRh_Rsp), *Starkeya novella* (BPrAlRhXa_Sno), *Xanthobacter autotrophicus* (BPrAlRhXa_Xau), *Roseovarius nubinhibens* (BPrAlRh_Rnu), *Roseovarius sp. TM1035* (BPrAlRh_Rsp_14), *Roseobacter sp. AzwK-3b* (BPrAlRh_Rsp_16), *Rhodobacter sphaeroides* (BPrAlRh_Rsp_4), *Roseovarius sp. 217* (BPrAlRh_Rsp_5), *Rhodobacter sphaeroides* (BPrAlRh_Rsp_8), *Methylovorus* (BPrBeMe_Mgl), *Neisseria elongata* (BPrBeNe_Nel), *Neisseria lactamica* (BPrBeNe_Nla), *Nitrosomonas sp. AL212* (BPrBeNi_Nsp), *Methylobacillus flagellatus* (BPrBeMe_Mfl), *Pelobacter carbinolicus* (BPrDeDe_Pca), *Psychrobacter sp. PRwf-1* (BPrGaPs_Psp_2), *Halomonas* (BPrGaOc_Hsp), *Acinetobacter* (BPrGaPs_Ara_1), *Acinetobacter* (BPrGaPs_Asp), *Psychrobacter sp. 1501* (BPrGaPs_Psp_1)

Actinobacteria (22): *Propionibacterium avidum* (BAcAcAc_Pav), *Propionibacterium acnes* (BAcAcAc_Pac__2), *Propionibacterium acnes* (BAcAcAc_Pac), *Propionibacterium freudenreichii* (BAcAcAc_Pfr), *Microlunatus phosphovorus* (BAcAcAc_Mph), *Bifidobacterium longum* (BAcAcBi_Blo__2), *Bifidobacterium longum* (BAcAcBi_Blo__1), *Bifidobacterium longum* (BAcAcBi_Blo), *Bifidobacterium breve* (BAcAcBi_Bbr), *Bifidobacterium bifidum* (BAcAcBi_Bbi__1), *Bifidobacterium dentium* (BAcAcBi_Bde__1), *Bifidobacterium adolescentis* (BAcAcBi_Bad), *Streptomyces sp. C* (BAcAcAc_Ssp_8), *Streptomyces sp. Mg1* (BAcAcAc_Ssp_6), *Streptomyces sp. e14* (BAcAcAc_Ssp_4), *Streptomyces coelicolor* (BAcAcAc_Sco_1), *Streptomyces violaceusniger* (BAcAcAc_Svi_2), *Streptomyces himastatinicus* (BAcAcAc_Shi), *Streptomyces bingchenggensis* (BAcAcAc_Sbi), *Frankia sp. EUN1f* (BAcAcAc_Fsp__3), Frankia sp. EAN1pec (BAcAcAc_Fsp), *Frankia alni* (BAcAcAc_Fal)
