# Supplementary material for: Long-Term Persistence of Bi-functionality Contributes to the Robustness of Microbial Life through Exaptation
Source: PLoS Genet. 2016 Jan 29;12(1):e1005836. doi: 10.1371/journal.pgen.1005836 (PMC4732765; doi:10.1371/journal.pgen.1005836)
Supplement: S2 Table — (PDF) [file pgen.1005836.s004.pdf]

S2 Table. Nucleotide and amino acid sequences of synthesized genes for ancestral proteins

Nucleotide sequence for CA-Act-HisA (restriction sites for *Sph*I and *Hind*III are underlined):

GCATGCTCACCCTGCTGCCTGCAGTTGATGTTCGTGATGGTCAGGCAGTTCGTCTGGTTCAGGGTGAA
GCAGGTAGCGAAACCAGCTATGGTGATCCGCTGGAAGCAGCACGTACCTGGCAAGAGGATGGTGCAGA
ATGGATTCATCTGGTTGATCTGGATGCAGCATTTGGTCGTGGTAGCAATCGTGAACTGATTGCCGAAG
TTGTTCGTGCCGTTGATGTTAATGTTGAACTGAGCGGTGGTATTCGTGATGATGATAGCCTGGATGCC
GCACTGGCAACCGGTGCAGCACGTGTTAATATTGGCACCGCAGCACTGGAAAATCCGGAATGGGTTCG
TAAAGTTATTGATCGTTATGGCGATCGTATTGCAGTTGGTCTGGATGTGCGTGGCACCACCCTGGCAG
CCCGTGGTTGGACCCGTGATGGTGGTGAACTGTTTGAAGTTCTGGCACGTCTGGATGCGGCAGGTTGT
GCACGTTATGTTGTTACCGATGTTGCACGTGATGGTATGCTGACCGGTCCGAATGTGGAACTGCTGCG
TGAAGTTACCGCAGCAACCGATCGTCCGGTTGTTGCAAGCGGTGGTGTTAGCAGTCTGGATGATCTGC
GTGCACTGGCAGCGCTGGTTCCGGAAGGTGTTGAAGGTGCAATTGTTGGTAAAGCACTGTATGCCGGT
GCATTTACCCTGCCGGAAGCACTGGCCGTTGCACGTAAGCTT

Nucleotide sequence for CA-Prot-HisA (restriction sites for *Sph*I and *Hind*III are underlined):

GCATGCTGATTATTCCGGCAATCGATCTGAAAGATGGTCGTTGTGTTCGTCTGGAACAGGGTGATATG
GAAAAAGCAACCGTGTATAATGATGATCCGGCAGCAATGGCACGTCAGTGGGTTGAGCAGGGTGCAGA
ATGGCTGCATCTGGTTGATCTGGATGGTGCATTTGCAGGTAAACCGGTTAATGAAGATGCCATTAAAG
CAATTGCAGAAGCAGTTAGCATTCCGGTTCAGCTGGGTGGTGGTATTCGTGATCTGGAAACCATTGAA
GCATATCTGGAAGCAGGTATTGATCGTGTTATTATTGGCACCGTTGCAGTGAAAAATCCGGAACTGGT
TCGTGAAGCATGTCGTGCATTTCCGGGTCGTATTGTTGTTGGTATTGATGCACGTGATGGTATGGTTG
CAGTTAAAGGTTGGGCAGAAGTTACCGAAGTTAAAGCCACCGATCTGGCCAAACGTTTTGAAGATGCG
GGTGTTGAAGCAATCATTTATACCGATATTGCCCGTGATGGCATGATGCAGGGTCCGAATATTGAAGC
AACCCGTGCACTGGCAAAAGCAGTTTCAATTCCGGTTATTGCAAGCGGTGGTGTTAGCAGCCTGGAAG
ATATCGAAGCACTGCTGGCAATTGAAGATAGCGGTGTGACCGGTGTTATTACCGGCAAAGCACTGTAT
GAAGGTAGCCTGGATCTGCGTGAAGCACTGGCACTGGCCAAAAAGCTT

Nucleotide sequence for CA-Bact-HisA (restriction sites for *Sph*I and *Hind*III are underlined):

GCATGCGCATTATTCCGGCAATCGATCTGAAAGATGGTCGTTGTGTTCGTCTGGTTCAGGGTGATATG
GAAAAAGCAACCGTGTATAATGATGATCCGCTGGAAATGGCAAAACAGTGGGTTGAACAGGGTGCAGA
ATGGCTGCATGTTGTTGATCTGGATGGTGCATTTGCAGGTAAACCGGTTAATGAAGATGTGATCAAAG
AAATCGCACAGAAAGTTAGCGTTAAAGTTCAGCTGGGTGGTGGTATTCGTGATCTGGAAGATATTGAA
GCATATCTGGATGCCGGTGTTGATCGTGTTATTATTGGCACCGTTGCAGTTAAAAATCCGGAACTGGT
TCGTGAAATGGTGGAAAAATATGGTGAACGTATTGTGGTTGGTATTGATGCACGTGATGGTATGGTTG

CCGTTAAAGGTTGGAAAGAAACCACCGAAGTTAAAGCCACCGATCTGGCCAAACGTTTTGAAGATGCA
GGCGTTGAAGCAATCATTTATACCGATATTGCCCGTGATGGCATGATGCAGGGTCCGAACATTGAAGC
CATTCGTGAACTGGCAAAAGCAGTTAGCCTGCCGGTTATTGCAAGCGGTGGTGTTAGCAGCCTGGAAG
ATATCGAGGCACTGCTGGCAATTGAAGAAAGCGGTGTTGCGGGTGTTATTGTTGGTAAAGCACTGTAT
GAAGGTCGTCTGGATCTGCGTGAAGCACTGGCACTGGCCAAAAAGCTT

Amino acid sequence of CA-Act-HisA:

MLTLLPAVDVRDGQAVRLVQGEAGSETSYGDPLEAARTWQEDGAEWIHLVDLDAAFGRGSNRELIAEV
VRAVDVNVELSGGIRDDDSLDAALATGAARVNIGTAALENPEWVRKVIDRYGDRIAVGLDVRGTTLAA
RGWTRDGGELFEVLARLDAAGCARYVVTDVARDGMLTGPNVELLREVTAATDRPVVASGGVSSLDDLR
ALAALVPEGVEGAIVGKALYAGAFTLPEALAVAR

Amino acid sequence of CA-Prot-HisA:

MLIIPAIDLKDGRCVRLEQGDMEKATVYNDDPAAMARQWVEQGAEWLHLVDLDGAFAGKPVNEDAIKA
IAEAVSIPVQLGGGIRDLETIEAYLEAGIDRVIIGTVAVKNPELVREACRAFPGRIVVGIDARDGMVA
VKGWAEVTEVKATDLAKRFEDAGVEAIIYTDIARDGMMQGPNIEATRALAKAVSIPVIASGGVSSLED
IEALLAIEDSGVTGVITGKALYEGSLDLREALALAK

Amino acid sequence of CA-Bact-HisA:

MRIIPAIDLKDGRCVRLVQGDMEKATVYNDDPLEMAKQWVEQGAEWLHVVDLDGAFAGKPVNEDVIKE
IAQKVSVKVQLGGGIRDLEDIEAYLDAGVDRVIIGTVAVKNPELVREMVEKYGERIVVGIDARDGMVA
VKGWKETTEVKATDLAKRFEDAGVEAIIYTDIARDGMMQGPNIEAIRELAKAVSLPVIASGGVSSLED
IEALLAIEESGVAGVIVGKALYEGRLDLREALALAK
