# Supplementary material for: Long-Term Persistence of Bi-functionality Contributes to the Robustness of Microbial Life through Exaptation
Source: PLoS Genet. 2016 Jan 29;12(1):e1005836. doi: 10.1371/journal.pgen.1005836 (PMC4732765; doi:10.1371/journal.pgen.1005836)
Supplement: S3 Table — (PDF) [file pgen.1005836.s005.pdf]

S3 Table. List of oligonucleotides used for cloning and site-directed mutagenesis

**Name**	**Sequence**
**5`dd*his*A_*Nde*I**	ccgtatcatatgattatttttcccgctgttg
**3`dd*his*A_*Xho*I**	agccccctcgaggcgctttcgggcgtgtttttc
**5`dd*his*A_*Sph*I**	ggagatggcatgctgatttttcccgctg
**3`dd*his*A_Stopp_*Hind*III**	tgccaagcttcaggcgctttcgggcgtg
**5`dd*his*A_C516T**	attgaacgcgacgg**t**atgcagtgcggc
**3`dd*his*A_C516T**	gccgcactgcat**a**ccgtcgcgttcaat
**5`pc*his*A_*Nde*I**	ccgtatcatatgatagttattcccgccatcg
**3`pc*his*A_*Xho*I**	agccccctcgaggctttgcccttggtcagagcc
**5`pc*his*A_*Sph*I**	ggagatggcatgctggttattcccgcca
**3`pc*his*A_Stopp_*Hind*III**	tgccaagcttcaggctttgcccttggt
**5`tm*his*A_*Nde*I**	ccgtatcatatgctcgttgtcccggcgat
**3`tm*his*A_*Not*I**	atagcggccgcgcgagcatatctcttcatcac
**5`tm*his*A_*Sph*I**	atagcatgctcgttgtcccggcg
**3`tm*his*A_Stopp_*Hind*III**	tgccaagctttagcgagcatatct
**5`sc*pri*A_*Sph*I**	ggagatggcatgcgcaagctcgaactc
**3`sc*pri*A_ Stopp_*Hind*III**	tgccaagcttcacgacgtagcctccaa
**5`pET24a_*Nde*I_to_*Sph*I**	ccaccagtcatgctagcc**gc**atgcatatctccttcttaaag
**3`pET24a_*Nde*I_to_*Sph*I**	ctttaagaaggagatatgcat**gc**ggctagcatgactggtgg
**5`pET24a_A536T**	cgccatctccttgc**t**tgcaccattccttg
**3`pET24a_A536T**	caaggaatggtgca**a**gcaaggagatggcg

Restriction sites are underlined; modified bases are in bold.
